# Wire Electrochemical Micro Machining of Narrow Slits on Fe-Based Amorphous Alloys

**DOI:** 10.3390/mi17101143

**Published:** 2026-09-30

**Authors:** Yusen Hang, Tao Yang, Xiujuan Wu, Weijing Kong

**Affiliations:** 1 College of Mechanical Engineering, Nanjing University of Industry Technology, Nanjing 210023, China; 2 Jiangsu Provincial Key Laboratory of Precision and Micro-Manufacturing Technology, Nanjing University of Aeronautics and Astronautics, Nanjing 210016, China; 3 Precision Manufacturing Engineering and Technology Research Center of Jiangsu Province, Nanjing University of Industry Technology, Nanjing 210023, China

**Keywords:** wire electrochemical micromachining, Fe-based amorphous alloy, electrochemical characteristics, narrow-slit process optimization, microstructure

## Abstract

Amorphous alloys (metallic glasses) possess unique long–disordered but short–ordered atomic structures and outstanding mechanical properties, including high hardness, high strength, and good corrosion resistance, making them attractive for precision microdevice applications. However, their poor machinability limits their practical deployment because conventional thermomechanical processing can readily cause crystallization-related defects due to heat input. Wire electrochemical micromachining (WECM), based on anodic dissolution, generates no cutting heat or tool electrode wear and thus prevents crystallization damage during the fabrication of complex microstructures. This study systematically explores the electrochemical dissolution behavior and narrow-slit machining performance of Fe-based amorphous alloys. Slit-cutting experiments are conducted to reveal the effects of key parameters, including electrolyte type, feed rate, and electrical parameters, on machining characteristics. Following single-factor experimental investigation and parameter selection, 0.2 mol/L sulfuric acid is selected as the optimal electrolyte. The optimal parameters are a machining voltage of 7 V, a pulse width of 200 ns, a feed rate of 0.2 μm/s, and a wire electrode vibration amplitude of 100 μm. Uniform narrow slits with good surface quality are achieved. Representative complex microstructures are successfully fabricated. This work verifies the feasibility of WECM for the microfabrication of Fe-based amorphous alloys and advances its potential applications in microelectromechanical systems (MEMS) and aerospace components.

## 1. Introduction

With continuous trends toward higher precision, miniaturization, and superior performance in advanced manufacturing, metallic micro-slit structures have become essential components of core devices in the aerospace, national defense, biomedical engineering, and precision communication industries. Their machining accuracy, edge quality, and structural integrity directly determine the service performance and competitiveness of end-use equipment [1,2,3,4,5]. Conventional mechanical cutting, laser machining, and electrical discharge machining inevitably introduce burrs, residual stresses, heat-affected zones, microcracks, and recast layers during micro-slit fabrication. These drawbacks become particularly prominent for thermally sensitive, high-strength, and high-hardness metallic materials, limiting the performance and reliability of micro-components [6,7].

Wire electrochemical micro-cutting (WECM) removes material through electrochemical anodic dissolution. It is a noncontact specialized micromachining technique characterized by zero cutting force, negligible electrode wear, and damage-free processing without thermal effects [8]. When combined with nanosecond pulse power supplies, WECM substantially improves machining localization and enables high-accuracy micro-slit formation at the micron scale, attracting extensive research interest in the high-quality microfabrication of difficult-to-process, high-strength, thermosensitive materials [9,10].

Fe-based amorphous alloys are free of crystal defects such as grain boundaries and dislocations. They exhibit high hardness, excellent wear resistance and unique electrochemical passivation characteristics. Compared with Zr-based and Ni-based amorphous alloys, they feature much lower raw material cost and distinctive soft magnetic properties, showing promising application potential in MEMS microstructural components [11,12]. However, Fe-based amorphous alloys are extremely sensitive to processing temperatures, and conventional thermally related machining processes can readily trigger crystallization and substantially degrade material performance. Hence, the high-quality fabrication of micro-slits using conventional precision manufacturing methods remains challenging [13].

In WECM, workpiece material is removed in ionic form, ensuring high machining precision [14,15]. Compared to alternative micromanufacturing technologies, WECM is performed by anodic dissolution under near-room-temperature electrolyte environments. Compared with thermal machining methods, it can largely avoid recast layers and heat-affected zones owing to its low macroscopic thermal input, which helps alleviate thermal-triggered crystallization-related degradation of amorphous alloys. It should be noted that complete elimination of residual stress and tool electrode consumption cannot be achieved in real-world machining. These features render WECM well-suited for the fabrication of amorphous-alloy microstructures [16,17,18]. Cole et al. [19] from the University of Toronto observed the primary corrosion mechanisms during electrochemical micromachining through micromachining microscopic characterization. They determined super passivation potentials via chronoamperometry to suppress spontaneous passivation and optimized the electrolyte flow rate, duty cycle, and pulse voltage ranges. Koza et al. [20] at the Leibniz Institute for Solid State and Materials Research Dresden investigated the electrochemical micromachining of Zr-based amorphous alloys using microtool electrodes and highlighted the significance of electrolyte selection. Their experiments demonstrated the superior performance of methanolic perchloric acid solutions, with micro-holes having a depth of ~40 μm and an aspect ratio of 1 successfully machined. Sueptitz et al. [21] at Ruhr Universität Bochum fabricated microgrooves in amorphous alloys using sub-micro-second pulses and methanolic sulfuric acid electrolytes. They analyzed the effects of pulse width and pulse voltage on machining gaps and surface morphology and verified the feasibility of electrochemical micropatterning of amorphous alloys. Meng et al. [22,23,24] at Nanjing University of Aeronautics and Astronautics performed wire electrochemical micromachining on thin-sheet Ni-based amorphous alloys and achieved micropattern formation. A 10 µm diameter wire electrode was adopted, and microstructures were successfully fabricated on Ni-based amorphous alloys using 0.1 M HCl electrolyte at an applied voltage of 4.5 V, pulse duration of 80 ns, pulse period of 3 µs, and a feed rate of 0.3 µm/s. Current research on WECM of amorphous alloys mainly focuses on Zr-based and Ni-based systems, while studies concerning WECM of Fe-based amorphous alloys remain relatively scarce. The alloy composition leads to markedly different electrochemical passivation behavior from that of Zr- and Ni-based amorphous alloys. Investigating its WECM forming rules is valuable for expanding the manufacturing technology of microstructures made from iron-based amorphous alloys.

However, WECM investigations specifically targeting Fe-based amorphous alloys remain at an exploratory stage. The existing literature mainly focuses on feasibility demonstrations and preliminary process investigations. Systematic studies of micro-slit forming quality, slit width uniformity, minimum slit width control, processing stability, and complex structure adaptability remain insufficient. The mechanisms by which electrolyte composition, electrical parameters, feed strategies, and wire vibration conditions influence slit morphology, dimensional accuracy, and surface integrity have not been fully clarified. Reproducible and well-controlled narrow optimization protocols are also lacking, which limits the ability of current processes to satisfy the stringent dimensional consistency requirements of high-end microdevices.

Accordingly, this study presents a systematic investigation of WECM narrow-slit formation in Fe-based amorphous alloys. Electrolyte systems were screened, and the effects of machining voltage, pulse width, feed rate, and wire vibration parameters on slit quality were investigated. A single-factor experimental investigation and parameter selection strategy was established to achieve precise dimensional control and high-fidelity slit fabrication. Representative complex microstructures were fabricated for experimental validation. This work provides practical process guidelines for the scalable, high-precision fabrication of micro-slits in Fe-based amorphous alloys and raises their engineering applications in advanced manufacturing.

## 2. Materials and Methods

**Figure 1:**
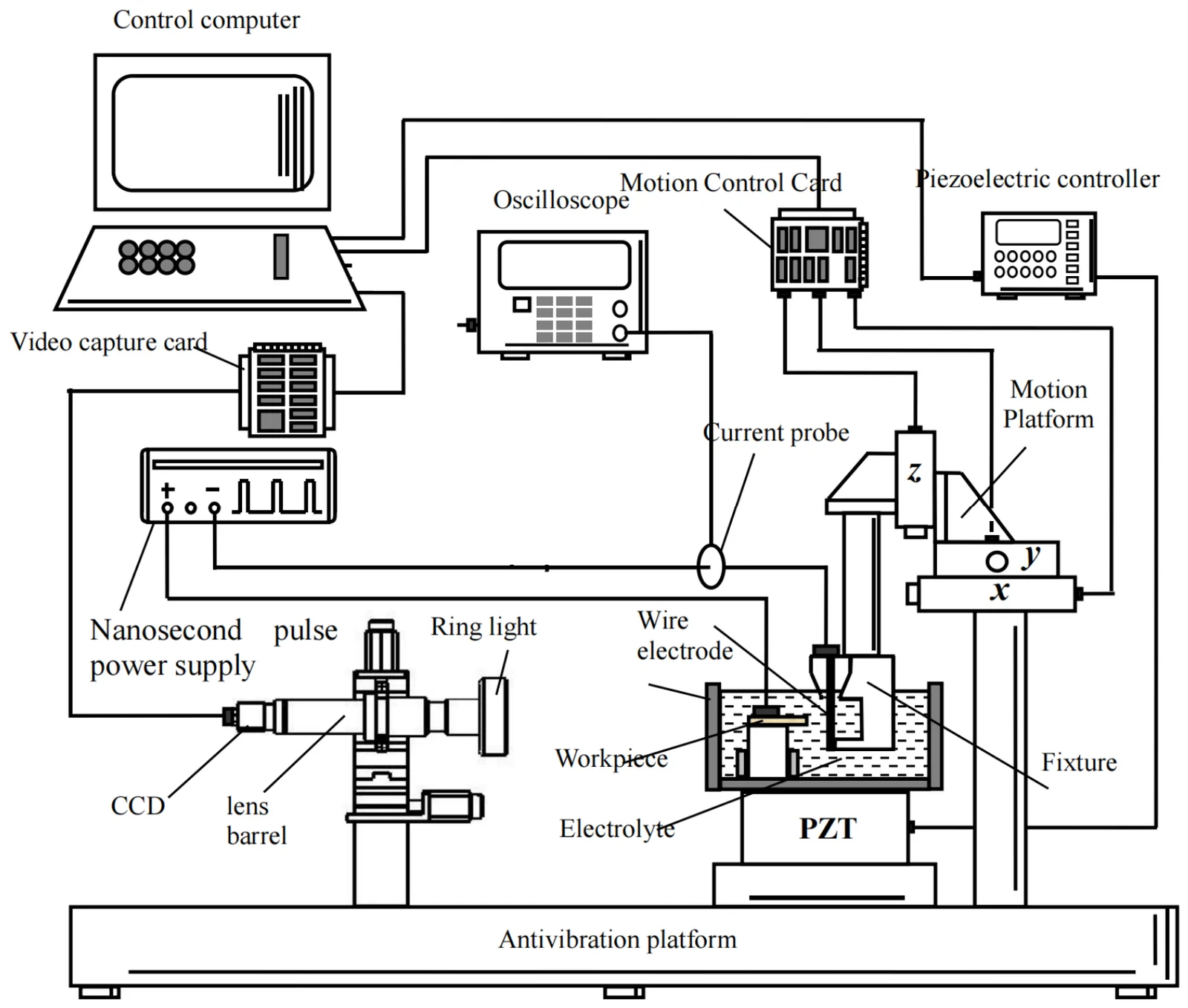
Schematic diagram of the self-built wire electrochemical micro-cutting experimental platform.

Figure 1 illustrates the material removal principle of WECM. When immersed in the electrolyte, the wire electrode is fed toward the workpiece and selectively dissolves the metallic substrate through anodic reactions. The pulsed power supply delivers a well-regulated current to ensure uniform and controlled material dissolution. The synergistic reciprocating vibration and linear feeding of the wire electrode continuously expand the machined area, enabling complex contour cutting. The wire electrode performs axial reciprocating sinusoidal vibration, and all amplitude values reported in this paper represent peak-to-peak amplitude. The entire process generates negligible mechanical force and preserves the dimensional accuracy of thin-walled and fragile micro-components.

**Figure 2:**
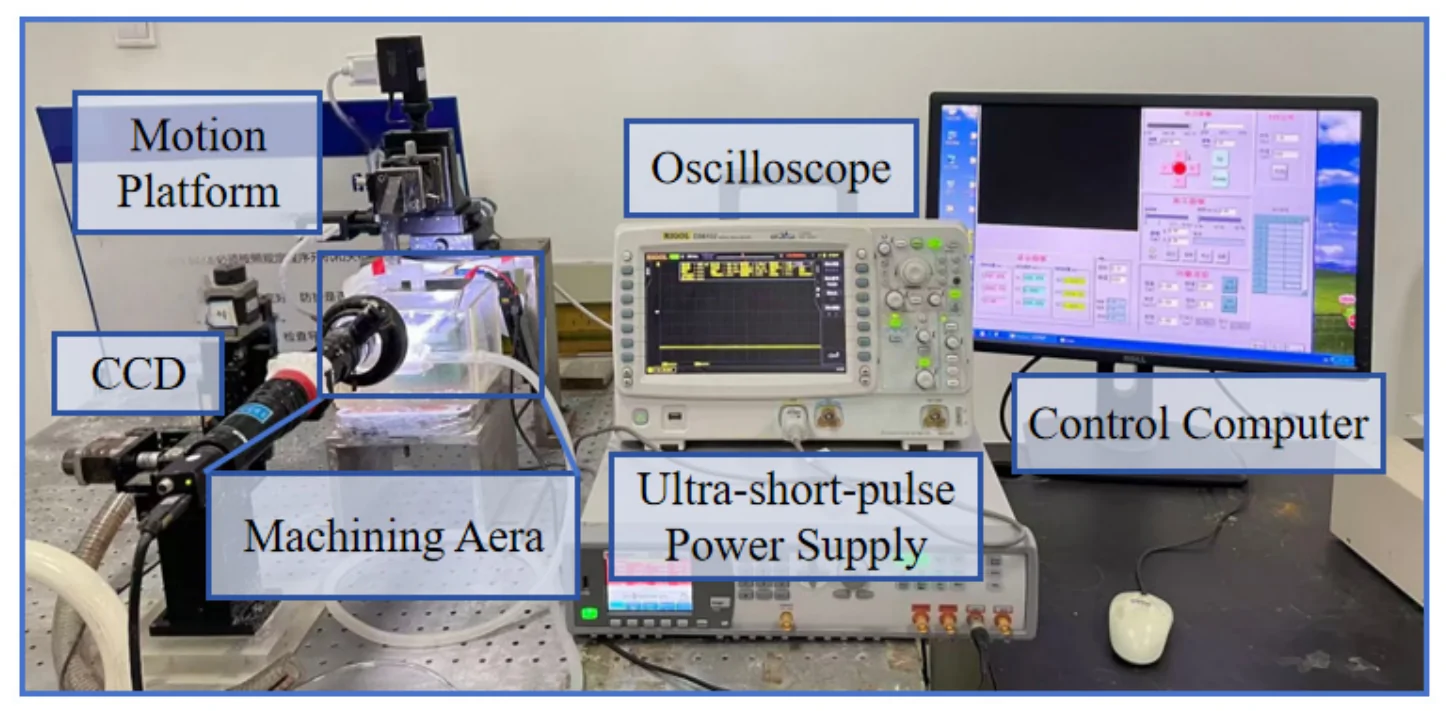
Photograph of the experimental setup.

All experiments were conducted using a self-developed WECM platform, as shown in Figure 2. The experimental setup mainly comprises a nanosecond pulse power supply (81104A, Agilent, Santa Clara, CA, USA), a high-precision three-axis motion stage (M406.DG, PI, Karlsruhe, Germany), a wire electrode tensioning and axial vibration module (P158.ZCD, PI, Karlsruhe, Germany), an electrolyte circulation system, a Charge-Coupled Device (CCD) online observation subsystem, a current detection unit, and an oscilloscope (DS6104, RIGOL, Suzhou, China). This platform supports submicrometer feeding resolution, nanosecond adjustment, and low-frequency vibration control to meet the requirements of high-accuracy micro-slit machining.

The material used in this work is Fe_48_Ni_31_Co_1_C_8_B_12_ amorphous alloy, and the listed composition is given in atomic percent. Fe-based amorphous alloy foils with a thickness of 100 μm were used as workpieces. Tungsten wires with a diameter of 10 μm were selected as wire electrodes because of their high stiffness, excellent electrical conductivity, and corrosion resistance during microelectrochemical machining. Four candidate electrolytes (NaCl, NaNO_3_, HCl, and H_2_SO_4_ aqueous solutions) were compared to evaluate their electrochemical performance. The initial inter-electrode gap is set to 5 μm before formal cutting. After tensioning, the tension of the tungsten wire electrode is approximately 0.27 N, and the unsupported wire length is about 10 mm. All machining experiments adopt the immersion electrolyte mode without forced jet flushing. The wire electrode performs axial reciprocating sinusoidal vibration driven by piezoelectric ceramics. The feed cutting-path length for each individual micro-slit is around 200 μm. The main experimental parameters adopted are summarized in Table 1.

Experiments were conducted to investigate the effects of machining voltage, pulse width, feed rate, vibration amplitude, and vibration frequency on slit width, width uniformity, and edge morphology. Slit width measurements were performed using digital microscope (DVM5000, Leica, Wetzlar, Germany; VH-Z500, Keyence, Osaka, Japan). Each machining condition was repeated five times independently to ensure the reliability of the experimental results. For each machined micro-slit, five measurement positions were evenly arranged along the full slit length, covering the near-entry region, three intermediate positions, and the exit region. The width of the micro-slit at each position was measured, and the average slit width of the individual specimen was determined from these five measurements. On this basis, the mean slit width under each parameter condition was obtained from the five repeated experiments, and the corresponding standard deviation was calculated to characterize the machining stability and data dispersion. All results reported in terms of mean and standard deviation in this paper follow the above measurement and evaluation procedure.

## 3. Results and Discussion

### 3.1. Electrochemical Characteristics of the Fe-Based Amorphous Alloy

**Figure 3:**
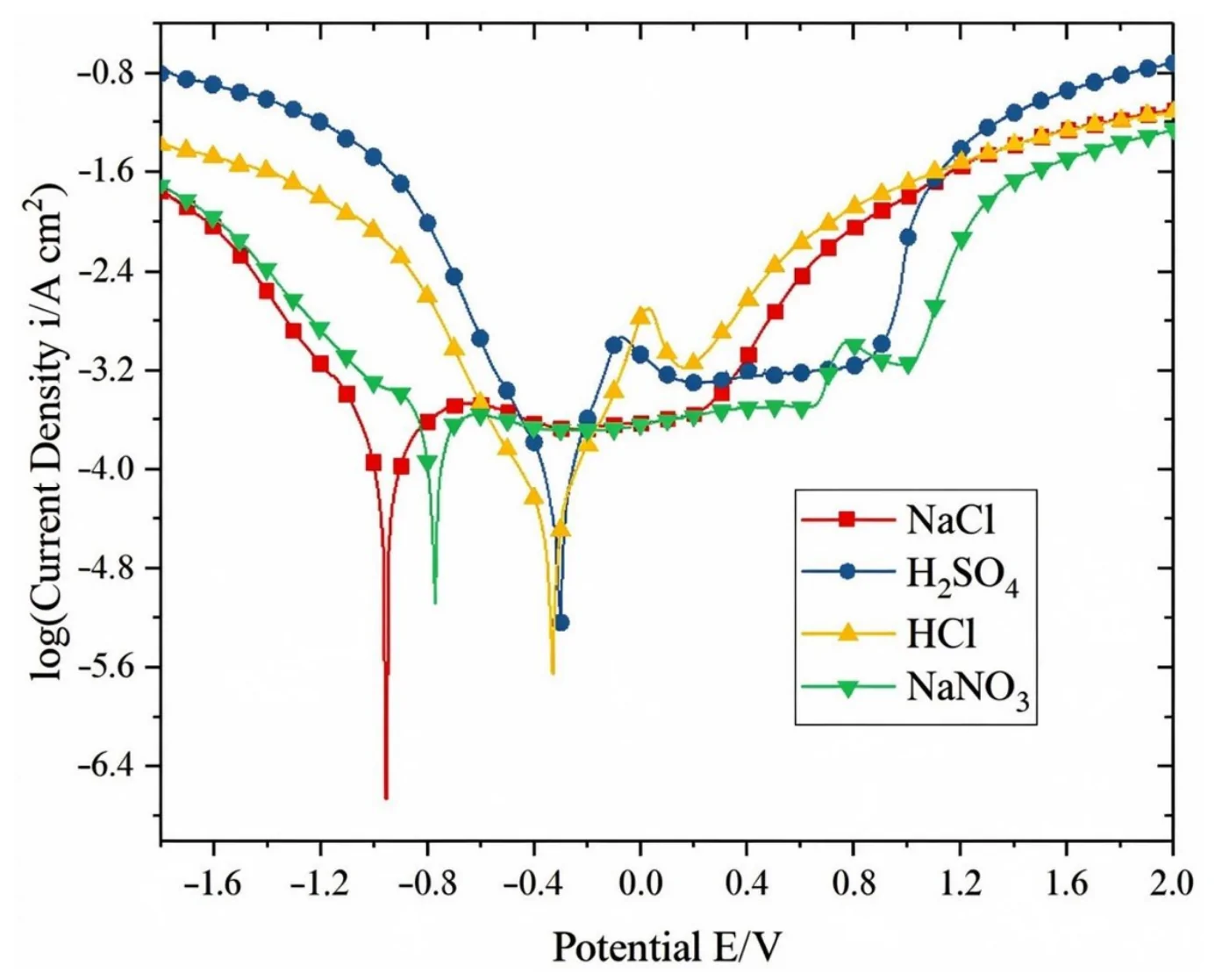
Polarization curves of Fe-based amorphous alloy in different electrolytes.

Potentiodynamic polarization curves of Fe-based amorphous alloys in four different electrolytes are illustrated in Figure 3. Typical activation, passivation, and superpassivation behaviors can be observed for all measured solutions. Four candidate electrolytes including NaCl, NaNO_3_, HCl and H_2_SO_4_ aqueous solutions were adopted for polarization tests, and all electrolytes were prepared at the identical concentration of 0.2 mol/L.

Polarization measurements were performed on an electrochemical workstation (Zennium, Zahner, Kronach, Germany). The working electrode was a 100 μm thick Fe-based amorphous alloy sheet with an effective exposed area of approximately 100 mm^2^. A platinum foil and a saturated calomel electrode (SCE) were used as the counter and reference electrodes, respectively. All tests were performed at room temperature with repeated measurements to ensure data reliability.

Combining the polarization measurements with the experimental observations, the key electrochemical parameters are summarized as follows. In the NaCl solution, the corrosion potential is −0.95 V, the passivation current density is 1.5 μA/cm^2^, and the passivation potential window ranges from −0.75 to 0.26 V. In the NaNO_3_ solution, the corrosion potential is −0.76 V, the passivation current density ranges from 1.2 to 7.2 μA/cm^2^, and the incomplete passivation potential interval ranges from −0.65 to 1.02 V. In the HCl solution, the corrosion potential is −0.36 V, the passivation current density varies from 4.5 to 90 μA/cm^2^, and the incomplete passivation potential interval ranges from 0.02 to 0.96 V. In the H_2_SO_4_ solution, the corrosion potential is −0.32 V, the relatively stable passivation current density is 4.0 μA/cm^2^, and the passivation potential window ranges from −0.06 to 0.89 V.

According to the potentiodynamic polarization results, the Fe-based amorphous alloy possesses a relatively stable and intact passivation potential window in H_2_SO_4_, which facilitates the generation of compact passive film. The compact passive film is beneficial to suppress excessive stray etching and improve slit edge quality. In comparison, NaCl and NaNO_3_ exhibit low anodic dissolution activity and tend to induce frequent short-circuit risk during WECM. HCl displays unstable passivation behavior with obvious current fluctuation, forming loose passive film and leading to uncontrolled stray dissolution. Based on above-mentioned polarization characteristics, 0.2 mol/L H_2_SO_4_ was selected as the working electrolyte for subsequent systematic machining experiments.

### 3.2. Effect of Machining Voltage on Narrow-Slit Performance

**Figure 4:**
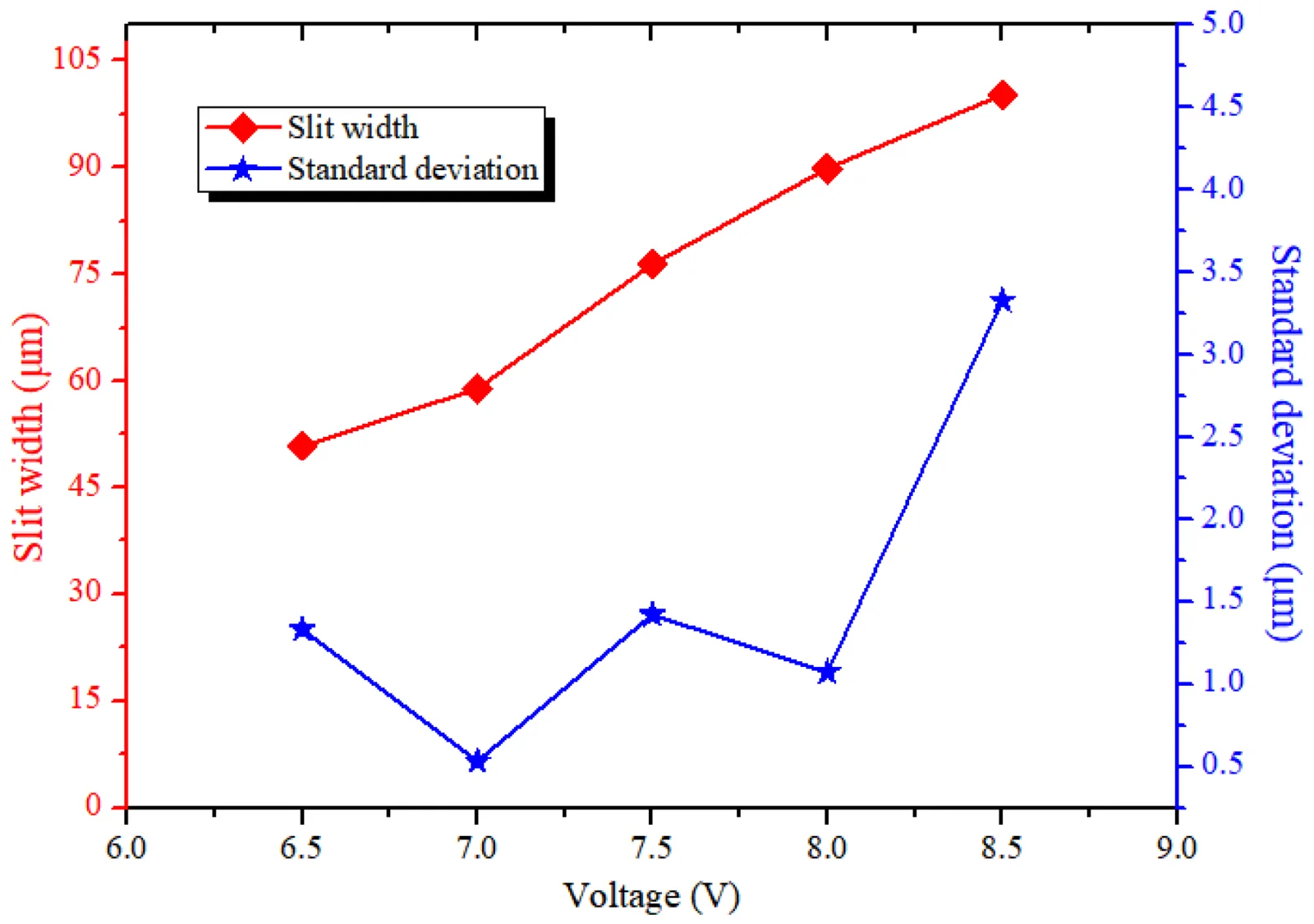
Variations in slit width and SD as a function of machining voltage.

**Figure 5:**
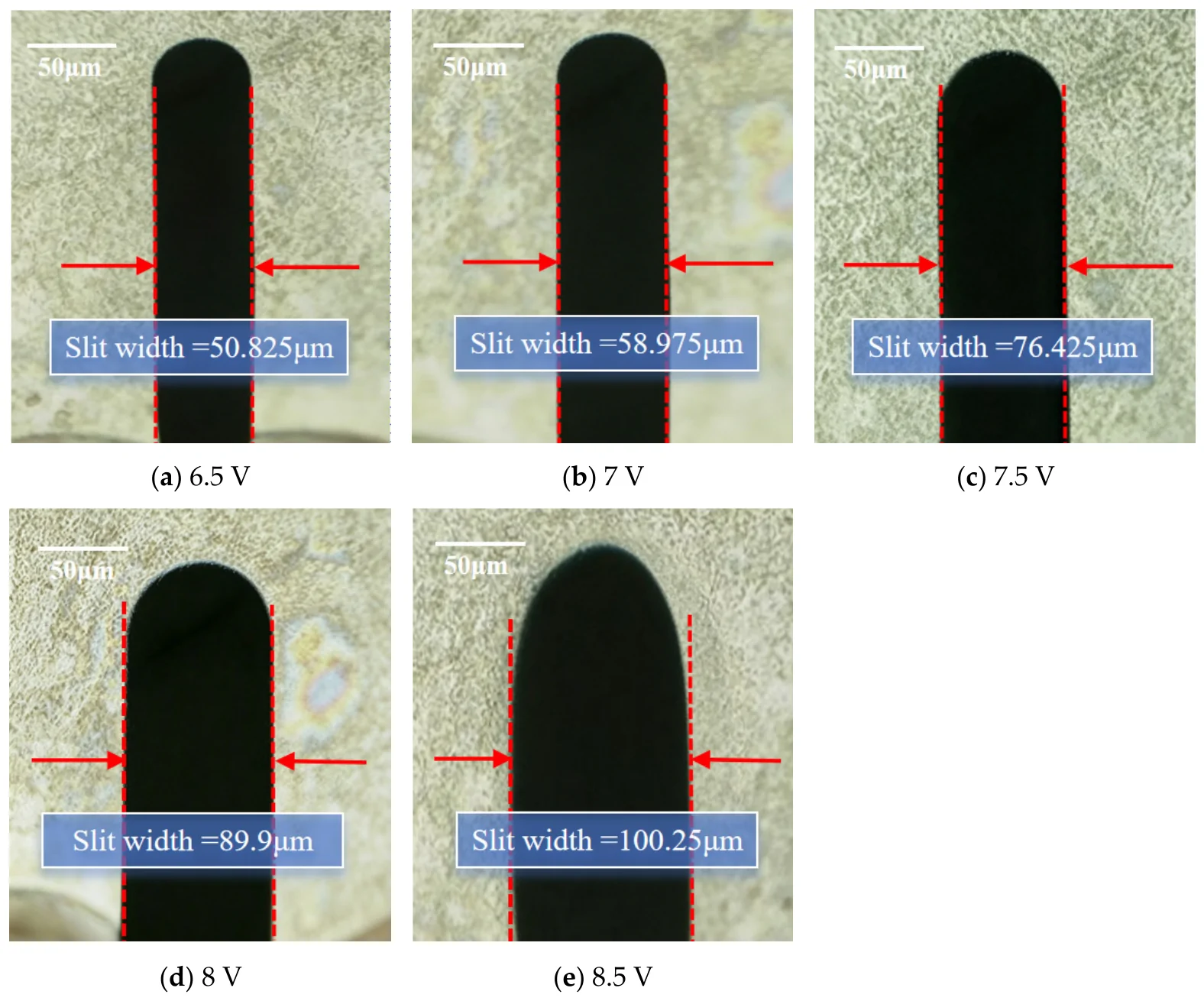
Micro-graphs of slits machined under different voltages: (**a**) 6.5 V; (**b**) 7.0 V; (**c**) 7.5 V; (**d**) 8.0 V; (**e**) 8.5 V.

A tungsten wire electrode with a diameter of 10 μm, a 100 μm thick Fe-based amorphous alloy workpiece, and a 0.2 mol/L H_2_SO_4_ electrolyte were employed. The fixed initial parameters were a pulse width of 250 ns, a pulse period of 3 μs, a wire electrode vibration amplitude of 200 μm, and a feed rate of 0.2 μm/s. The machining voltage was varied from 6.5 to 8.5 V. The slit width and standard deviation curves are illustrated in Figure 4, and the corresponding micro-slit micro-graphs are shown in Figure 5.

The slit width increases monotonically with increasing applied voltage. A higher voltage increases the electrochemical reaction current and material dissolution. Therefore, stray etching is intensified, and machining localization deteriorates, resulting in undesirable dissolution outside the target cutting zone and an increased slit width. The SD of the slit width reflects the processing stability and reaches its minimum value at 7 V, corresponding to the most uniform slit geometry. Considering the slit dimensions and consistency, 7 V is selected as the optimal machining voltage.

### 3.3. Effect of Pulse Width on Narrow-Slit Performance

**Figure 6:**
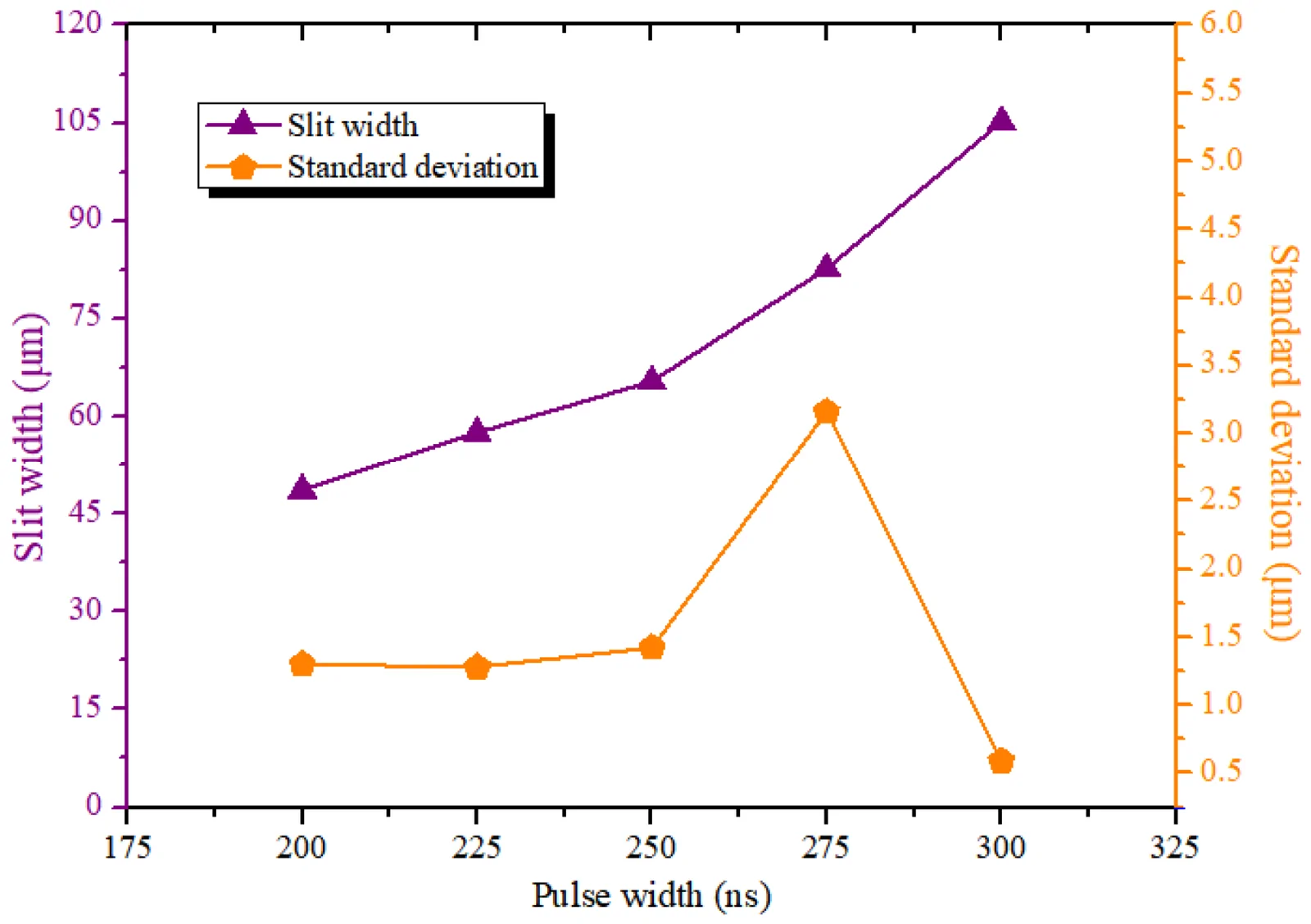
Slit width and SD against pulse width.

**Figure 7:**
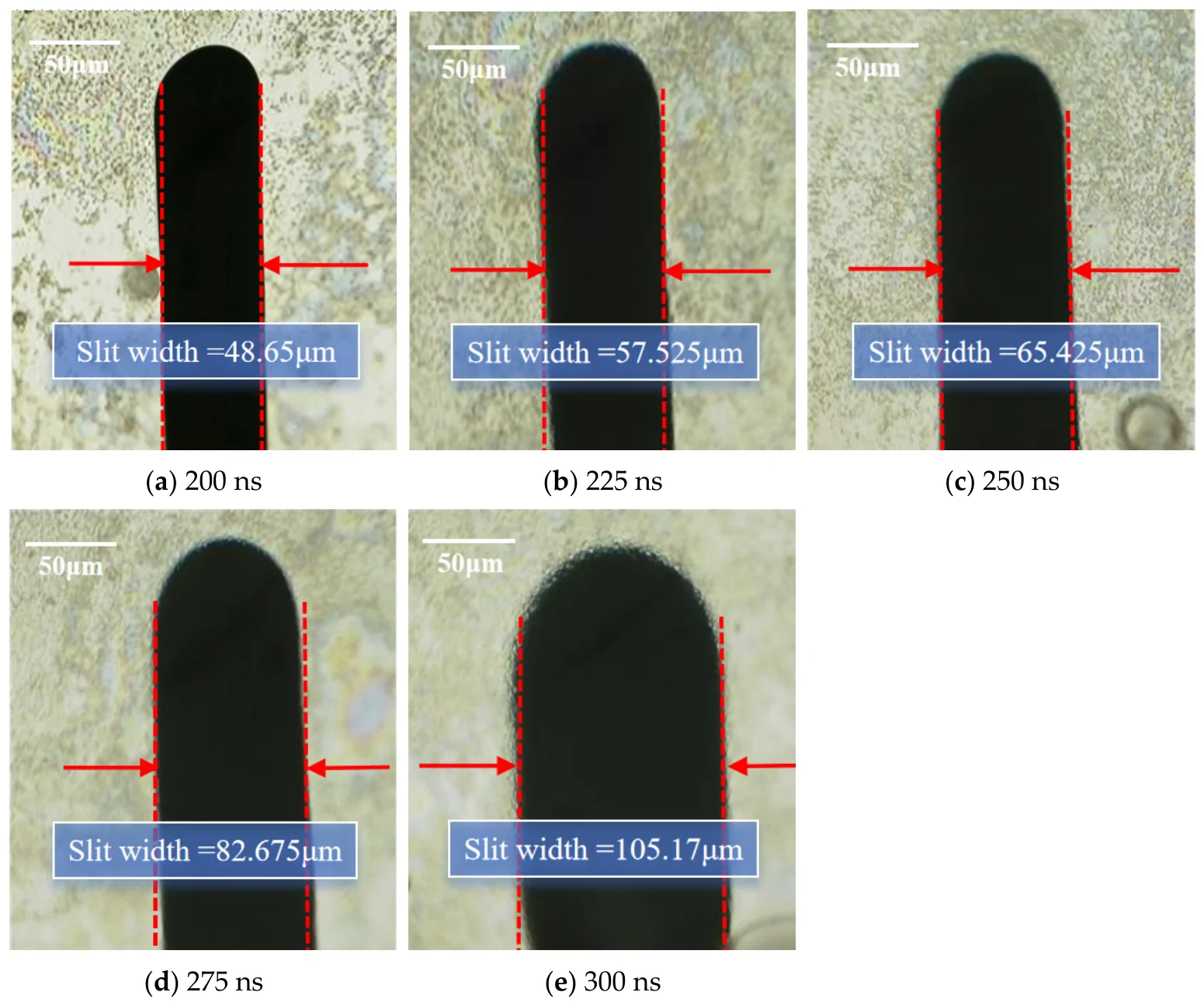
Micro-slit morphologies obtained at different pulse widths: (**a**) 200 ns; (**b**) 225 ns; (**c**) 250 ns; (**d**) 275 ns; (**e**) 300 ns.

The other experimental conditions were maintained at the same values as those described above. The pulse width was adjusted to 200, 225, 250, 275, and 300 ns. Figure 6 presents the slit width statistics, and Figure 7 shows the corresponding slit micro-graphs.

The slit width increases with increasing pulse width. A longer pulse duration extends the double-layer charging time and increases the electrode overpotential. Therefore, more regions exceed the critical reaction potential, the amount of material removal increases, the machining gap expands, and the localization capability deteriorates, broadening the slit. When the pulse width increases from 250 to 275 ns, the SD increases sharply, indicating deteriorated processing stability. Relatively low SD values and uniform slit profiles are maintained within the range of 200 to 250 ns. It should be noted that parameter selection is not only based on slit width standard deviation. Although 300 ns presents low standard deviation, it produces an excessively large slit width due to severe stray etching. Hence, 200 ns is selected as the preferred pulse width to balance slit dimension and dimensional uniformity.

### 3.4. Effect of Feed Rate on Narrow-Slit Performance

**Figure 8:**
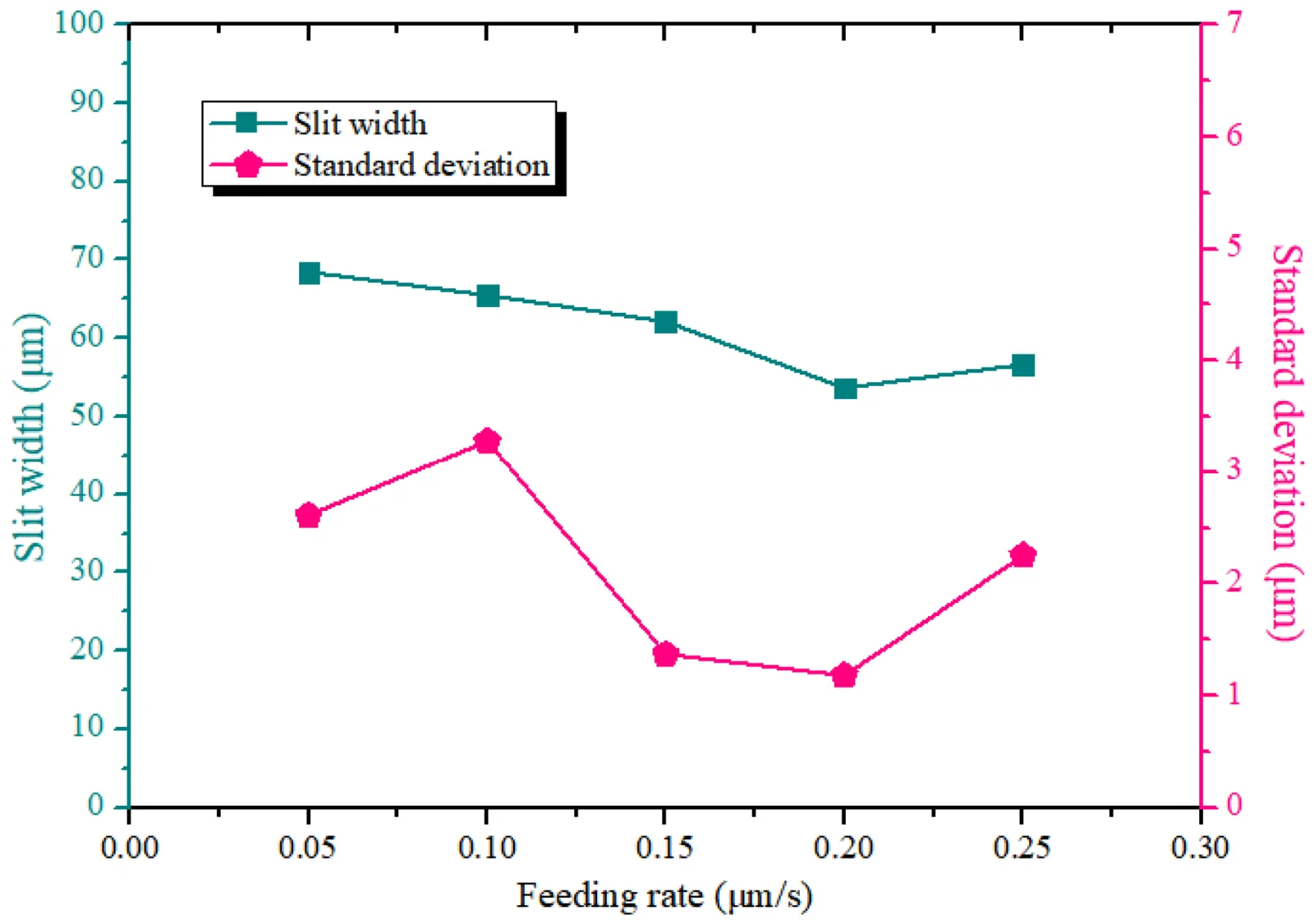
Slit width and SDSD as functions of feed rate.

**Figure 9:**
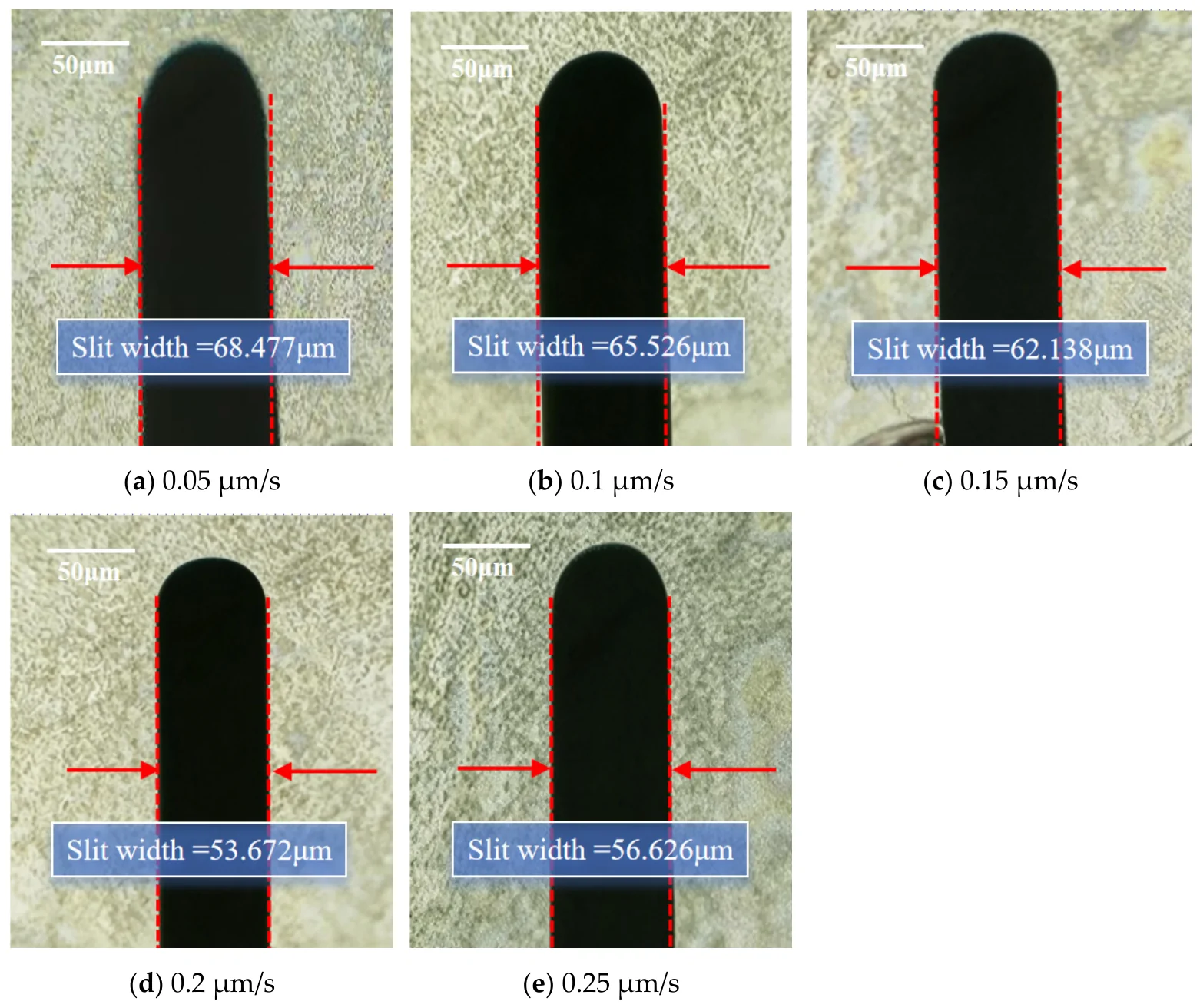
Micro-slit morphologies at different feed rates: (**a**) 0.05 μm/s; (**b**) 0.10 μm/s; (**c**) 0.15 μm/s; (**d**) 0.20 μm/s; (**e**) 0.25 μm/s.

Feed rates were set to 0.05, 0.10, 0.15, 0.20 and 0.25 μm/s, with other parameters unchanged. The statistical curves and slit micro-graphs are shown in Figure 8 and Figure 9.

The slit width decreases markedly with increasing elevated feed rate. A higher feed rate shortens the reaction duration of the wire electrode at each local position, reduces the effective dissolution area, and decreases material removal. However, an excessively high feed rate triggers intense electrochemical reactions and results in a slightly increased slit width. Therefore, the feed rate should be maintained within a reasonable range. The SD decreases significantly when the feed rate increases from 0.10 to 0.15 μm/s. Feed rates that are too low produce nonuniform slits, whereas the SD remains stable above 0.15 μm/s. A comprehensive evaluation indicates that 0.2 μm/s is the preferred feed rate.

### 3.5. Effect of Wire Electrode Vibration Amplitude on Narrow-Slit Performance

**Figure 10:**
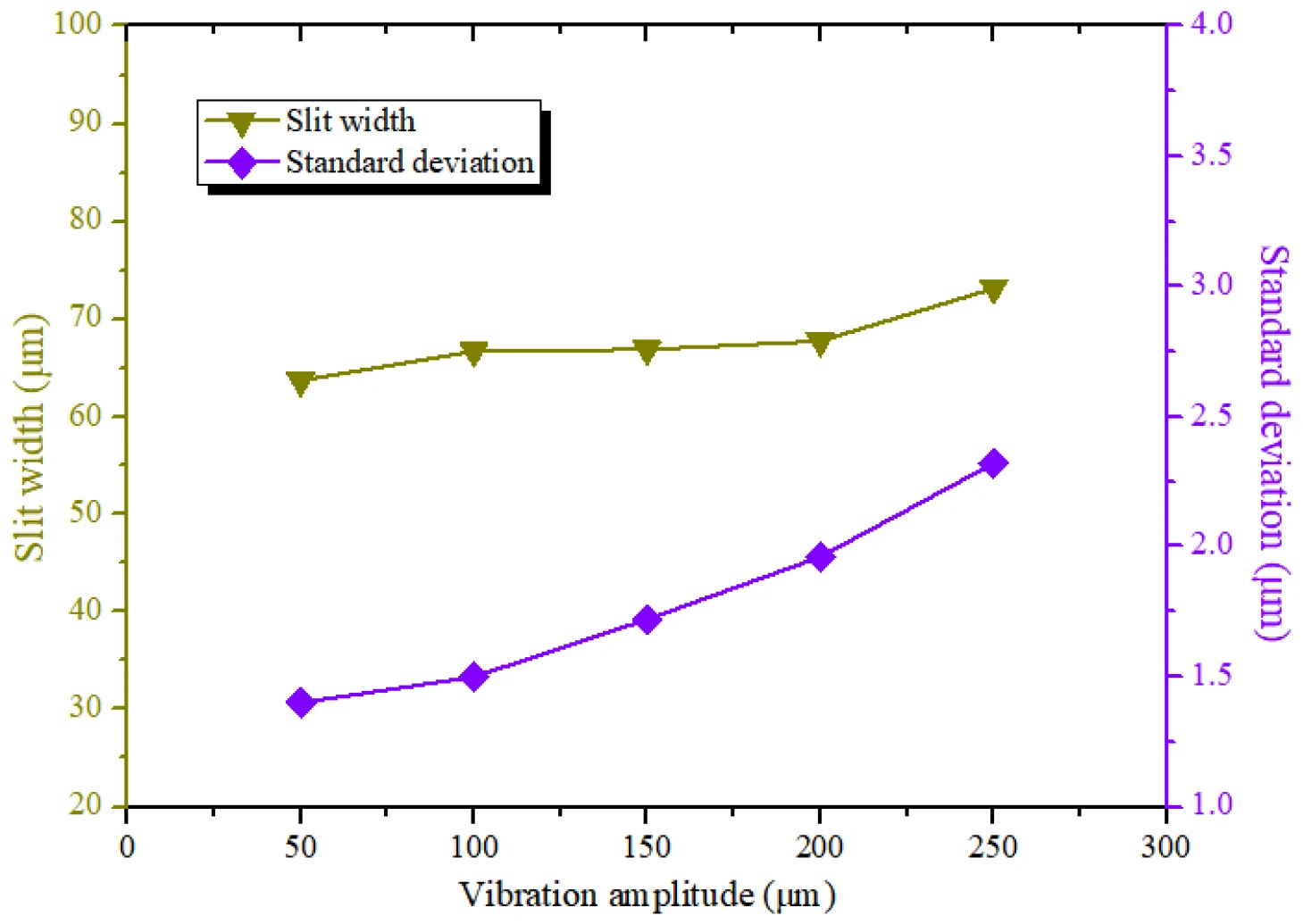
Slit width and SD versus wire electrode vibration amplitude.

**Figure 11:**
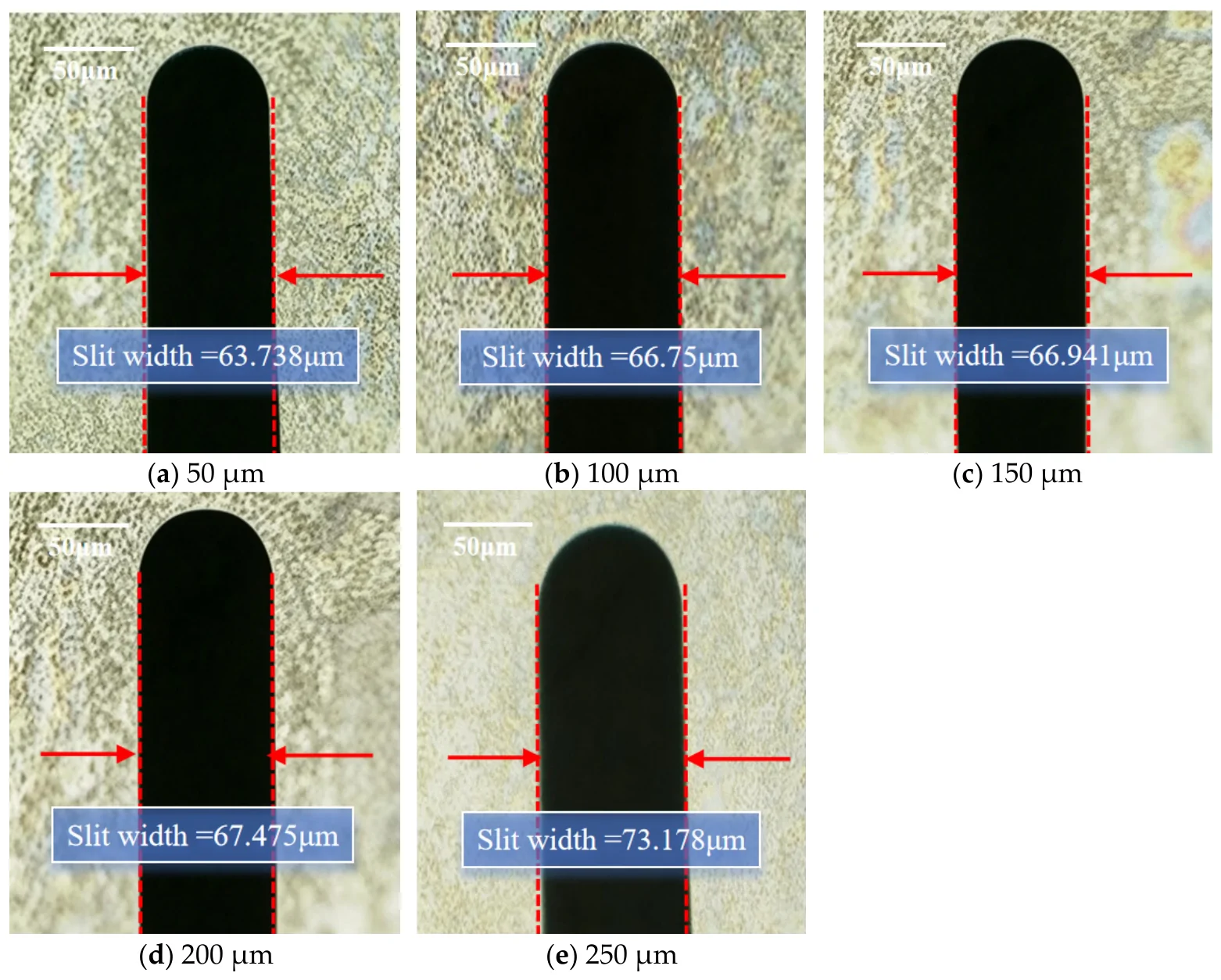
Slit morphologies under different wire electrode vibration amplitudes: (**a**) 50 μm; (**b**) 100 μm; (**c**) 150 μm; (**d**) 200 μm; (**e**) 250 μm.

Preliminary trials revealed that stable cutting cannot be sustained at 0.2 μm/s when vibration amplitude was fixed at 200 μm in this group of single-factor tests; accordingly, the feed rate was temporarily adjusted to 0.15 μm for this group of amplitude comparison experiments. When the vibration amplitude is optimized to 100 μm, stable continuous machining can be achieved at 0.2 μm/s for complex-structure fabrication. The vibration amplitude was set to 50, 100, 150, 200, and 250 μm. Figure 10 and Figure 11 present the experimental results.

The slit width varies moderately with the vibration amplitude. The narrowest slit is obtained at an amplitude of 50 μm, whereas the slit width changes only slightly within the amplitude range of 100 to 250 μm. However, the SD increases noticeably with increasing amplitude, indicating that the vibration amplitude has a more pronounced influence on machining stability than on slit width. The thin tungsten wire is flexible. Excessive vibration amplitude induces parasitic radial jitter beyond the intended axial motion and aggravates slit width fluctuation [25]. Although 50 μm vibration amplitude shows the minimum standard deviation in this single-factor test, small amplitude provides insufficient electrolyte flushing inside the narrow gap. It raises the risk of electrolytic product accumulation and short-circuit failure during long-time continuous cutting for complex contours. Consequently, 100 μm is selected, considering both dimensional consistency and gap-flushing stability for complex-structure fabrication.

### 3.6. Fabrication of Complex Microstructures Using Optimized WECM

**Figure 12:**
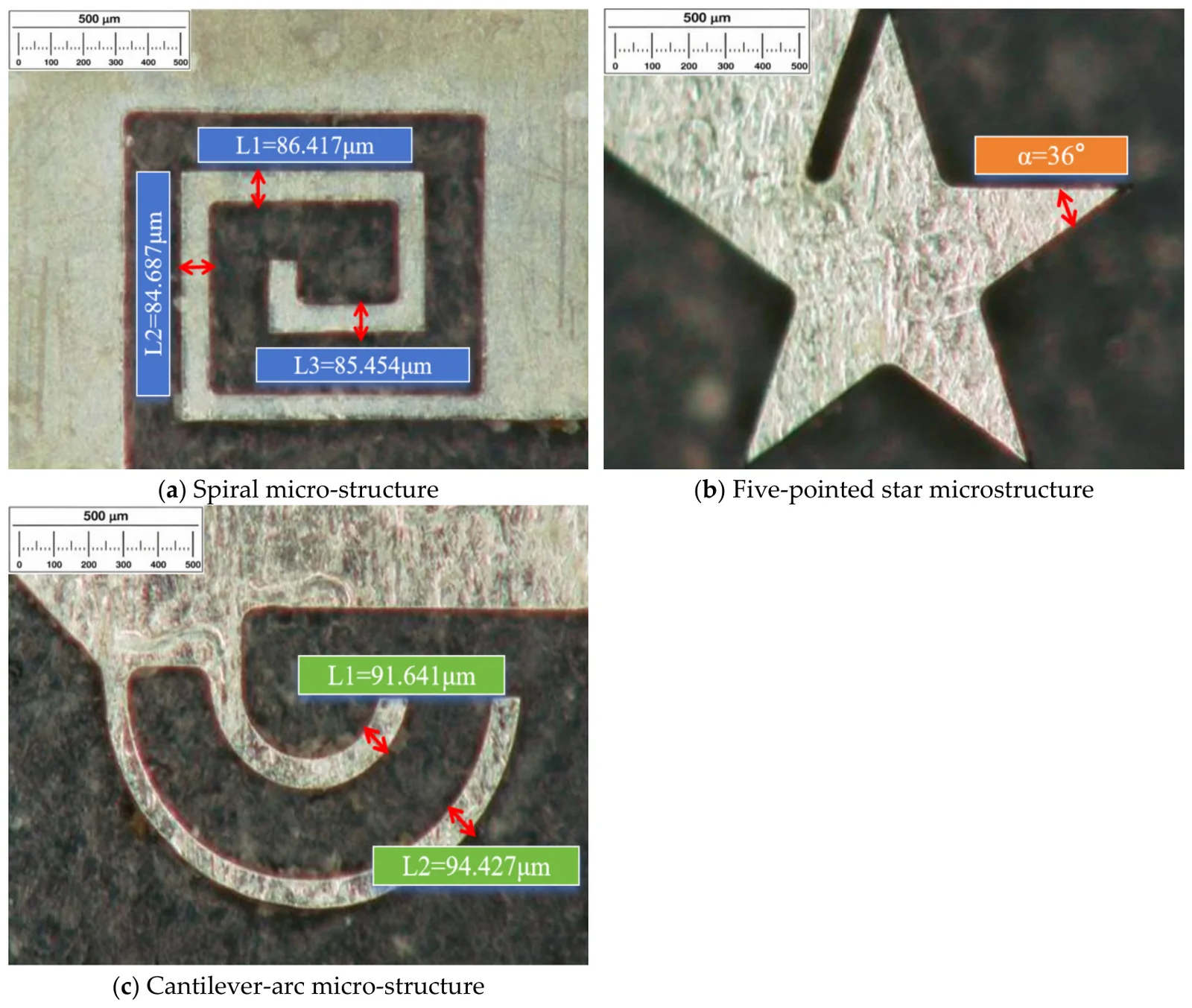
Complex micro-structures fabricated on Fe-based amorphous alloy by optimized WECM.

Complex microstructures were machined on Fe-based AA foils in 0.2 mol/L H_2_SO_4_ using the optimized parameters: a machining voltage of 7 V, a pulse width of 200 ns, a pulse period of 3 μs, a feed rate of 0.2 μm/s, and a wire electrode vibration amplitude of 100 μm. The machined patterns are depicted in Figure 12.

For the spiral pattern shown in Figure 12a, the average structural width is 85.519 μm, with an SD of 0.867 μm. The tip angle of the five-pointed star feature depicted in Figure 12b reaches 36°. The cantilever arc microstructure illustrated in Figure 12c has an average width of 93.034 μm and an SD of 1.97 μm. These results demonstrate that WECM can be utilized to fabricate not only delicate cantilever structures but also sharp angular contours, highlighting its considerable potential for the future manufacturing of miniature components.

## 4. Conclusions

This study systematically investigates wire electrochemical micro-cutting (WECM) for narrow-slit fabrication on Fe-based amorphous alloys. The main conclusions are summarized as follows:

(1) The electrochemical dissolution behaviors of Fe-based amorphous alloys in four different electrolytes are analyzed using potentiodynamic polarization tests. A 0.2 mol/L sulfuric acid aqueous solution is identified as the optimal working electrolyte.
(2) The slit width increases with increasing machining voltage and pulse width. As the feed rate increases, the slit width initially decreases and then increases marginally. A larger vibration amplitude aggravates wire electrode jitter, increases the standard deviation of the slit width, and reduces machining stability. The optimal processing parameters are as follows: machining voltage of 7 V, pulse width of 200 ns, feed rate of 0.2 μm/s, and wire electrode vibration amplitude of 100 μm. Uniform narrow slits with favorable surface quality are achieved under these conditions.
(3) Representative complex microstructures, including spiral patterns, five-pointed stars, and cantilever arcs, are successfully fabricated on Fe-based amorphous alloy foils under the optimized conditions. The resulting structures exhibit uniform dimensions, intact sharp corners, and damage-free cantilever features. These results validate the feasibility and reliability of WECM for the microfabrication of Fe-based amorphous alloys. This study provides practical process references for the high-precision fabrication of microstructures on Fe-based amorphous alloys and facilitates their applications in MEMS and aerospace microdevices.

## Figures and Tables

**Table 1:** Summary of WECM experimental parameters.

Parameter	Value
Workpiece material	Fe_48_Ni_31_Co_1_C_8_B_12_
Electrode diameter (µm)	10
Workpiece thickness (µm)	100
ECMM electrolyte type	NaCl, NaNO_3_, HCl, H_2_SO_4_
PC voltage (V)	6.5, 7.0, 7.5, 8.0, 8.5
Pulse period (µs)	3
Pulse width (ns)	200, 225, 250, 275, 300
Vibration amplitude (µm)	50, 100, 150, 200, 250
Vibration frequency (Hz)	1.5
Feed rate (µm/s)	0.05, 0.10, 0.15, 0.20, 0.25

## Data Availability

The original contributions presented in this study are included in the article. Further inquiries can be directed to the corresponding author.
